# Effects of Titanium Dioxide Nanoparticles and Coenzyme Q10 on Testicular Ischemia-Reperfusion Injury: Role of the Mitochondrial Apoptosis Pathway: The Effects of TiO2 NPs and CoQ10 on Testicular I/R Injury

**DOI:** 10.31661/gmj.v11i.2334

**Published:** 2022-12-31

**Authors:** Masoumeh Masoumi, Mitra Salehi, Seyed Abdolhamid Angaji, Mehrdad Hashemi

**Affiliations:** ^1^ Department of Infertility, Vali-E-Asr Reproductive Health Research Center, Family Health Research Institute, Tehran University of Medical Sciences, Tehran, Iran; ^2^ Department of Genetics, Faculty of Biosciences, Islamic Azad University, North Tehran Branch, Tehran, Iran; ^3^ Department of Microbiology, Faculty of Biosciences, Islamic Azad University, North Tehran Branch, Tehran, Iran; ^4^ Department of Cell and Molecular Biology, Faculty of Biosciences, Khwarizmi University, Tehran, Iran; ^5^ Department of Genetics, Faculty of Advanced Science and Technology, Islamic Azad University, Tehran Medical Sciences, Tehran, Iran; ^6^ Farhikhtegan Medical Convergence Sciences Research Center, Farhikhtegan Hospital Tehran Medical Sciences, Islamic Azad University, Tehran, Iran

**Keywords:** Testis, miR-21, circRNA0001518, Coenzyme Q10, Titanium Dioxide Nanoparticles

## Abstract

**Background:**

Testicular ischemia-reperfusion (I/R) injury is a urological emergency that can lead to male infertility. So far, no suitable treatment has been found for it. Therefore, in the present study, we investigate the therapeutic effects of concomitant administration of coenzyme Q10 (CoQ10) and titanium dioxide nanoparticles (TiO2-NPs) on testicular I/R damage in rats and the expressions of genes involved in mitochondrial apoptosis, miR-21, and circRNA0001518.

**Materials and Methods:**

In this study, after induction of testicular torsion/detorsion, CoQ10 and TiO2-NPs were administered to the rats for ten days. Then, sperm extracted from the epididymides were analyzed for concentration, viability, morphology, and motility. The amount of apoptosis in testicular cells was studied by flow cytometry. Also, the expressions of the Bax and Bcl-2 genes, as well as miR-21 and circRNA0001518 levels were evaluated.

**Results:**

Sperm parameters improved in the rats’ testicular that received CoQ10. Administration of TiO2-NPs to healthy rats increased apoptosis and the Bax/Bcl-2 expression ratio. However, its administration to testicular I/R rats alone or in combination with CoQ10 caused a decrease in apoptosis, the Bax/Bcl-2 expression ratio, and an increase in miR-21 and circRNA0001518 expressions.

**Conclusion:**

Overall, individual or joint administration ofTiO2-NPs or CoQ10 can have therapeutic effects on testicular I/R by altering the expressions of genes in the mitochondrial apoptotic pathway and their regulatory elements.

## Introduction

The testes develop in pairs in the posterior part of the peritoneum and posterior wall of the abdominal area and migrate during embryonic development. They eventually become suspended in the scrotum and at the end of the spermatic cord [1]. This organ has many twisted tubes, called seminiferous tubules, in which the sperm are made. The produced sperm are transferred to another tube, called the epididymis, where they are stored [2]. Male infertility depends on various parameters, such as the number, motility, viability, and morphology of the sperms, which are affected by several factors [3]. There are several reasons for insufficient blood supply to testicular tissue, which causes hypoxia and oxidative stress [4]. Reducing or blocking blood flow to the testicles increases the production of reactive oxygen species (ROS). ROS has a destructive effect on DNA and the function of proteins, leading to the peroxidation of membrane fatty acids [5]. Mammalian sperm are rich in unsaturated fatty acids that are sensitive to ROS assault, which subsequently reduces fertility [6]. Cell death due to an ischemic/reperfusion (I/R) injury is closely related to the production of free radicals and lipid peroxidation [7]. Although no definitive treatment has been found to prevent an I/R injury, some interventions to reduce this injury have recently been suggested. These include blocking the production of free radicals, using anti-inflammatory drugs and angiotensin-converting enzyme inhibitors, and substances such as adenosine, morphine, and statins [7]. The results of studies have shown that antioxidant and free radical scavenger compounds provide protective effects on I/R injury [8]. Coenzyme Q10 (CoQ10) is an important intracellular antioxidant found in all cell membranes [9]. Since CoQ10 is present in almost all ATP synthesis sites, it is called ubiquinone [10]. CoQ10 is chemically similar to vitamin K but is not considered a vitamin because it is synthesized in the body. CoQ10 acts as part of the electron transfer chain as an accepting unit for the transfer of electrons to oxygen and it plays an important role in ATP synthesis [10]. CoQ10 effectively inhibits the oxidation of lipids, proteins, and DNA, and intracellular regenerative systems continuously regenerate it. Ubiquinol is a reduced form of CoQ10 that can act as an important antioxidant in the protection of cell membrane molecules against oxidation [11]. Today, nanoparticles (NPs) are widely used in most sectors, including industry, agriculture, and medicine [12]. Titanium dioxide NPs (TiO2-NPs) have a wide range of applications due to their unique properties [12]. The effect of this NPs on various body processes has been observed. The results of studies show that low concentrations of NPs can have antioxidant effects and even prevent cell death; however, at high concentrations, they can increase apoptosis and cause oxidative stress [13][14]. The simultaneous application of NPs with an antioxidant compound appears to improve the antioxidant properties [15]. One of the factors involved in I/R damage are microRNAs (miRNAs) [16][17][18][19]. These small molecules (18-22 nucleotides) play an important role in the expression of genes involved in the I/R damage process [19]. The miR-21 plays a role in I/R damage and has been observed in hypoxic human renal epithelial cells [18]. miR-21 is referred to as an anti-apoptotic miRNA and it has a possible protective role in I/R damage [19]. The regulatory role of circular RNA (circRNAs) in a variety of physiological processes has been described. They seem to act as a miRNA sponge and cause change in gene expression [16]. These elements are also able to interact with and modulate protein activities [16]. Therefore, in recent years, much attention has been paid to the role of circRNAs in various cellular processes. In the present study, we assessed the effects of CoQ10 and TiO2-NPs on reducing I/R lesions of the testis and investigated the expressions of genes involved in mitochondrial apoptosis along with miR-21 and circ0001518.

## Materials and Methods

**Table:** Table1. Primer Sequences Used in the Current Study

**Genes**	**Sequence [3’-5’]**
***miR-21***	Ordered from Pars Zengan Company Code PG-1
***circRNA 0001518***	Ordered from Pars Zengan Company Code PG-1
***Bax***	F: AGGGTGGCTGGGAAGGC R:TGAGCGAGGCGGTGAGG
***Bcl-2***	F: ATCGCTCTGTGGATGACTGAGTAC R: AGAGACAGCCAGGAGAAATCAAAC
*β-Actin	F: CGGTTCCGATGCCCTGAGGCTCTT R: CGTCACACTTCATGATGGAATTGA

^*^Set as the internal control

### Animals

A total of 48 Wistar rats (250-300 g) at the age of 20 weeks were purchased from Pasteur Institute (Tehran, Iran) and exposed to a 12 hours’ light/dark cycle, 25 °C, and 50% relative humidity. All animals had free access to food and water, and the same proportions of corn, wheat, and barley were used to feed the rats. In order to avoid stress on animals and allow the rats to adapt to their environment, no experiments were performed on these rats for one week, and all experiments were conducted during 09:00 AM-15-00 PM.

### Induction of Testicle Torsion

After anesthetizing the rats with 10 mg/ml of ketamine (Panpharma, Germany) and xylazine 2% (20 mg/ml, Alfasan, the Netherlands), testicular torsion was performed in the experimental group by twisting the testicles for 720 degrees counterclockwise for 1.5 hours. Detorsion was continued for ten days, when the rats were treated with the intended material. Then, after pathological confirmation of severe oligoasthenoteratozoospermia, the rats were placed into eight groups. The groups were the same in terms of age and weight [17].

### Groups and Design

In this study, TiO2-NPs (purity: 99/98%, particle size: 21 nm, density 3.84) were obtained from Merck Corp (Germany). Injectable Q10 (CoQ10 red) was purchased from the Antiaging Institute (USA). We determined the dose of TiO2-NPs based on the median lethal dose (LD50), the concentration that caused the death of half of the rats. Accordingly, 0.005, 0.01, 0.02, 0.03, 0.04, and 0.05 mg/kg body weight concentrations were given to the rats. Based on the results, the LD50 was determined to 0.02 mg/kg body weight. The concentration of CoQ10 was the same as the concentration of NPs. The rats were randomly divided into the following eight groups (six rats per group):

1. Healthy rats (control)

2. Healthy rats that received TiO2-NPs

3. Healthy rats that received CoQ10

4. Rats that received simultaneous TiO2-NPs and CoQ10

5. Torsion/detorsion rats (no treatment)

6. Torsion/detorsion rats that received TiO2-NPs

7. Torsion/detorsion rats that received CoQ10

8. Torsion/detorsion rats that received TiO2-NPs and CoQ10.

### Sperm Analysis

First, all equipment related to sperm collection was heated to 37 °C with a hot plate. The rats were euthanized by a puncture injection of sodium thiopental, and their testicular tissues were isolated. The epididymides were removed under sterile conditions and placed in a Falcon tube that contained 5 mL Hanks' Balanced Salt Solution medium (Ariya Fan Varzan Co., Iran) with 5 mg/ml bovine serum albumin (Ariya Fan Varzan Co., Iran). The epididymides were subsequently cut into small pieces to allow for the removal of the sperm, and sperm were incubated for 20 minutes at 37 °C. Sperm (10 μL) were transferred to a hemocytometer, and we counted the diluted sperm under an optical microscope (Olympus, Japan) at 40× magnification. In order to calculate sperm viability, we removed 20 μl of the sperm solution and added 10 μl of 0.05% Eosin-Nigrosin (Farzaneh Arman Co., Iran) dye to it. After two minutes of incubation at room temperature, we observed the sperm under a light microscope at 100× magnification. The nonviable sperms turned pink due to the destruction of the plasma membrane of their heads, whereas live sperms did not stain. Sperm motility was assessed by an optical microscope (40× magnification). For this purpose, 10 μL of the sperm solution was put on a microscope slide, and the percentage of motility was calculated [18]. The standard Papanicolaou staining was used to evaluate sperm morphology [18], which was observed under an optical microscope (100× magnification). We evaluated 200 sperm for the presence of abnormal morphology, which was defined as sperm with two heads, large head, small head, round head, no acrosome, no head, long or short tail, no tail or twisted tail, and cytoplasmic diameter.

### Viability Assay

For this purpose, 5 mg of 3-(4,5-Dimethylthiazol-2-yl)-2,5-diphenyltetrazolium bromide (MTT; Sigma Aldrich, Germany) was dissolved in 1 ml of phosphate-buffered saline (PBS; Sigma Aldrich, Germany), and the solution was made at a concentration of 5 mg/ml and stored in the refrigerator for use. Then, 10 to 20 mg of testicular tissue was lysed in a PBS buffer by a homogenizer and centrifuged at 4 °C for 12 minutes at 12,000 rpm. Afterthat, 50 μl of MTT solution was added to the tubes. The tubes were then incubated for 2 hours at 37 °C. After 2 hours of incubation, 500 μm dimethyl sulfoxide (Merck, Germany) was added to each tube and shaken well to dissolve all formazone crystals. The solution was then transferred to a 96-well, and 1 hour later, the absorption was read at 560 nm, and the reference wavelength was 630 nm with calibration of 1.99 by a multi-plate device [18].

### Apoptosis Measurement

First, the testicular tissue fragments were digested enzymatically with collagenase enzyme for 4 hours at 37 °C. After inactivation with 3% fetal bovine serum (FBS; Ariya Fan Varzan Co., Iran) and centrifugation, the resultant cell suspension was filtered and poured into plates. Next, 10 ml of Dulbecco's Modified Eagle Medium (Geniran, Iran) that contained FBS, L-glutamine (2 mM), penicillin (100 U/ml), streptomycin (100 μg/ml), and 3% non-essential amino acids were added to the plates, and the plates were incubated at 37 °C and 5% CO2.

Flow cytometry was used to evaluate necrosis and apoptosis in testicular tissue cells. In this method, Annexin V-FITC staining (Sigma Aldrich, Germany) was used to show apoptosis, and simultaneous staining with propidium iodide (PI) as a marker was used to distinguish between necrosis and apoptosis. For this purpose, 100 μl of binding buffer was added to the testis cells treated with TiO2-NPs and Q10 at LD50 concentration after washing with PBS and centrifugation, then incubated with 5 μl Annexin V-FITC in the dark at 4 °C for 15 minutes. After washing and re-centrifugation, 10 μl of PI (10 ml/100 ml PBS) was added to the cell precipitate, and flow cytometry was performed using a Partec GmbH flow cytometer (Partec PA S, Germany) [18].

### RNA Extraction, cDNA Synthesis, and Real-Time Transcription Polymerase Chain Reaction (RT-PCR) 

According to the manufacturer's instructions, RNA was extracted from the testicular tissue by an RNA Extraction Kit (Yekta Tajhiz Azma, Iran). The quantity of the extracted RNAs was evaluated by NanoDrop (Artin Azma Mehr Co, Iran). A cDNA Synthesis Kit (Yekta Tajhiz Azma, Iran) was used for cDNA synthesis based on the manufacturer's instructions. Quantitative measurement of DNA was performed with a NanoDrop device. The primers for the Bax and Bcl-2 genes were designed [18]. The primer sequences are provided in Table-1. Also, miR-21 and circRNA0001518 primers have been purchased from Pars Zengan Company (Iran). The RT-PCR (ABI 7300; Applied Biosystems, USA) timing and temperature program began at 95 °C for 30 seconds for cDNA denaturation. In the next step, 40 cycles at 95 °C for 5 seconds and 60 °C for 31 minutes were performed, followed by cycles at 95 °C for 15 seconds, 60 °C for 30 seconds, and 95 °C for 15 seconds [18].

### Ethical Consideration

The present study was performed at the Faculty of Pharmacy of Shahid Beheshti University and Imam Khomeini Hospital in accordance with ethical principles, national norms, and standards for conducting medical research. The study was approved by the ethical committee of Islamic Azad University, North Tehran Branch (approval code: IR.IAU.TNB.REC.1399.024).

### Statistical Analysis

The significant differences among groups were evaluated by one-way analysis of variance (ANOVA) and Tukey’s test, using SPSS software version 22 (IBM SPSS Statistics for Windows, Version 22.0. Armonk, NY: IBM Corp). A P-value<0.05 was considered as a significant difference.

## Results

**Figure-1:**
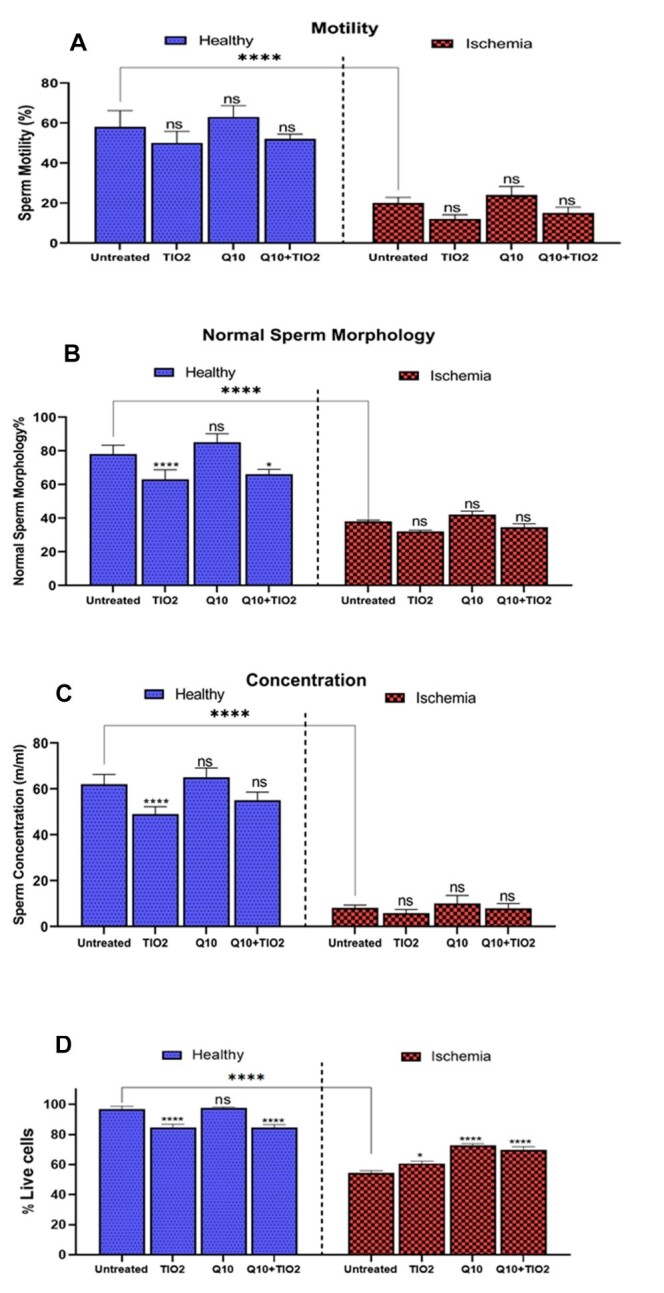
Figure 1. The effect of CoQ10 and TiO2-NPs on sperm motility (A), morphology (B), concentration (C), and viability (D) in different rat groups. * P<0.05.

**Figure-2:**
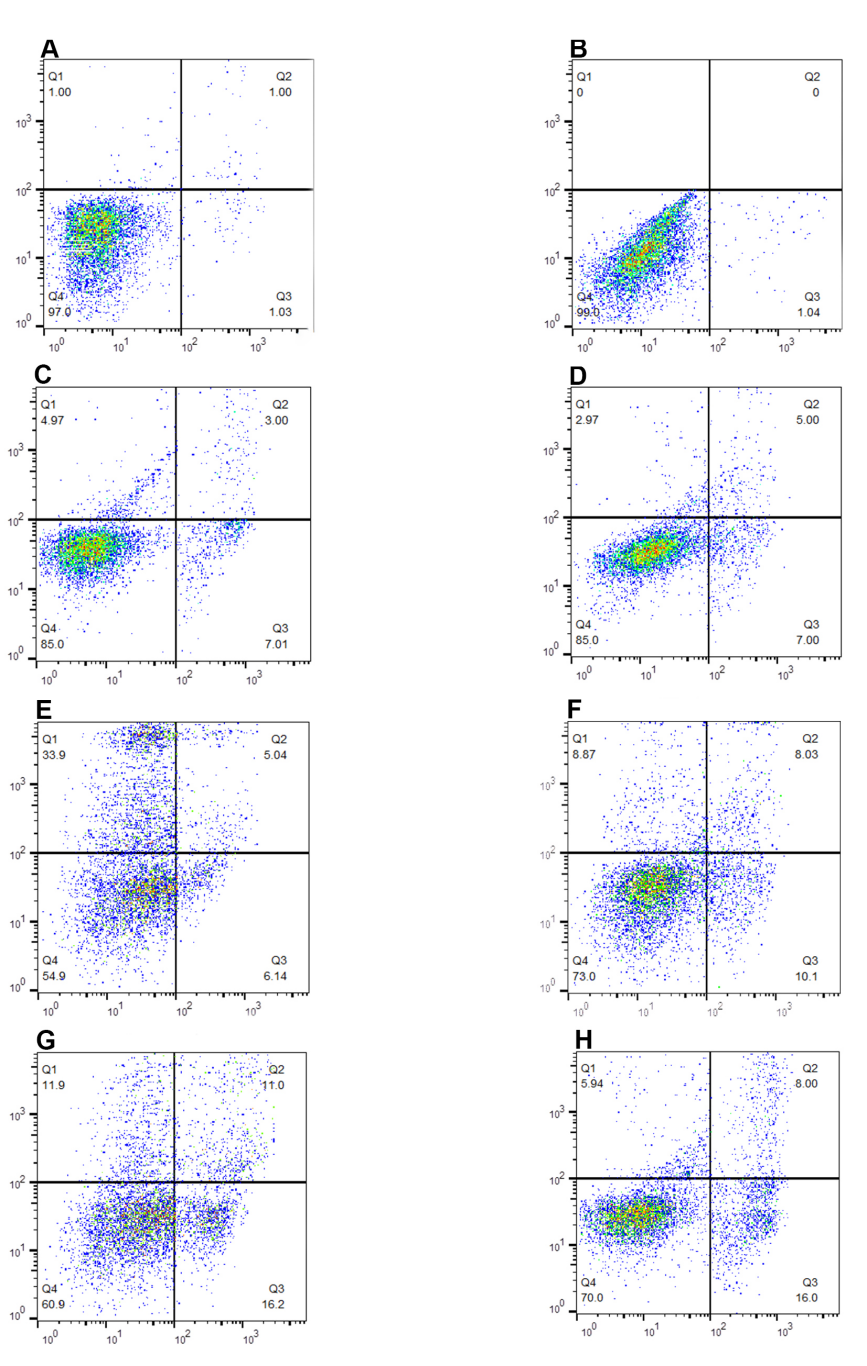
Figure 2. Flow cytometry analysis of apoptosis in testes cells of healthy rats (A) and rats in CoQ10 (B), TiO2-NPs (C), CoQ10+TiO2-NPs (D), I/R (E), I/R+CoQ10 (F), I/R+TiO2-NPs (G), and I/R+CoQ10+TiO2-NPs (H) groups.

**Figure-3:**
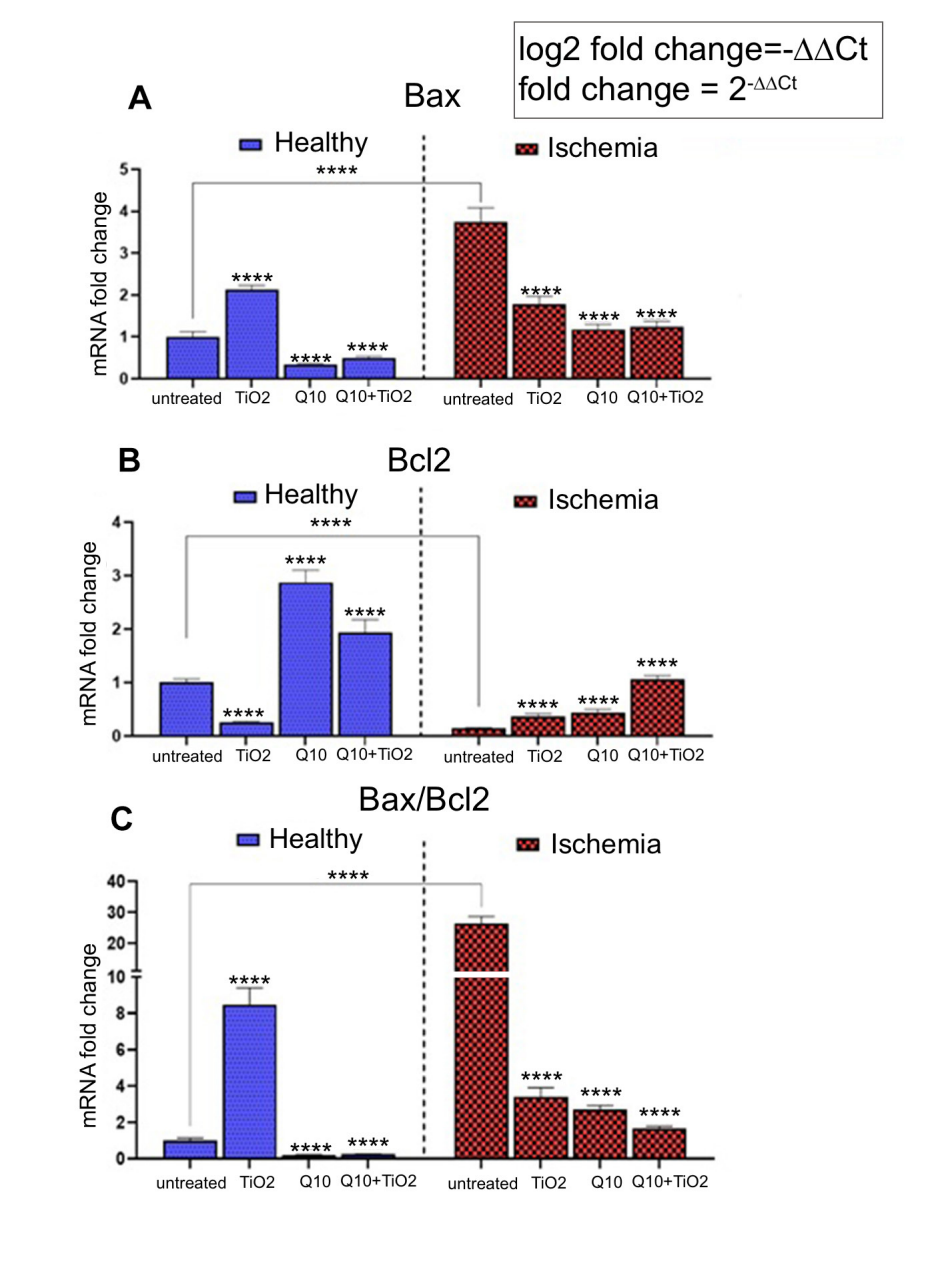
Figure 3. The expression levels of the Bax (A) and Bcl-2 (B) genes and the Bax/Bcl-2 ratio (C) in healthy and torsion/detorsion rats that received CoQ10+TiO2-NPs. **** P<0.0001 vs. untreated rats.

### Sperm Parameters

Induction of torsion/detorsion in the rat testes led to a sharp decrease in sperm motility, normal morphology, and concentration (Figure-1). Administration of TiO2-NPs to healthy and I/R rats reduced motility, normal sperm count, and sperm concentration compared to healthy controls. Also, in the healthy rats, CoQ10 administration improved sperm with normal morphology; however, in testicular I/R rats, an increase in all sperm parameters was observed with CoQ10 administration. Co-administration of TiO2-NPs with CoQ10 improved all sperm parameters in the testicular of I/R rats (Figure-1).

### Viability, Apoptosis, and Necrosis in Testicular Cells

The results showed a significant increase in the necrosis rate in the testicular cells of rats with testicular ischemia (P<0.001, Figure-2). However, TiO2-NPs decreased necrosis in the I/R group, whereas TiO2-NPs increased necrosis in the cells of the control healthy group. Also, CoQ10 showed a significant effect on reducing testicular cell necrosis after testicular torsion/detorsion (Figure-2). Co-administration of TiO2-NPs with CoQ10 reduced testicular cell death due to necrosis in the testicular ischemia group (P<0.001, Figure-2).

Flow cytometry results confirmed the increase in apoptosis in the healthy group and a decrease in apoptosis in the testicle I/R group after treatment with TiO2-NPs. Simultaneous treatment of TiO2-NPs with the CoQ10 reduced apoptosis (Figure-2).

### Genes Expression Levels

A comparison of Bax and Bcl-2 expression showed the lowest Bax and highest

Bcl-2 expressions in healthy rats that received CoQ10 (Figure-3). However, the highest Bax expression and the lowest Bcl-2 expression levels were seen in testicular I/R rats. The testicular torsion/detorsion significantly upregulated Bax and downregulated Bcl-2 expression levels. Treatment of healthy rats with TiO2-NPs caused overexpression of the Bax and downregulation of Bcl-2 levels. Treatment of testicular I/R rats with TiO2-NPs downregulated Bax and upregulated Bcl-2 levels (Figure-3).

The highest ratio of Bax/Bcl-2 gene

expressions was seen in the testicular I/R group, and the lowest was observed in the normal group that received CoQ10 (Figure-3). Treatment with TiO2-NPs decreased the Bax/Bcl-2 expression ratio in the testicular I/R rats and increased it in the normal healthy rats.

**Figure-4:**
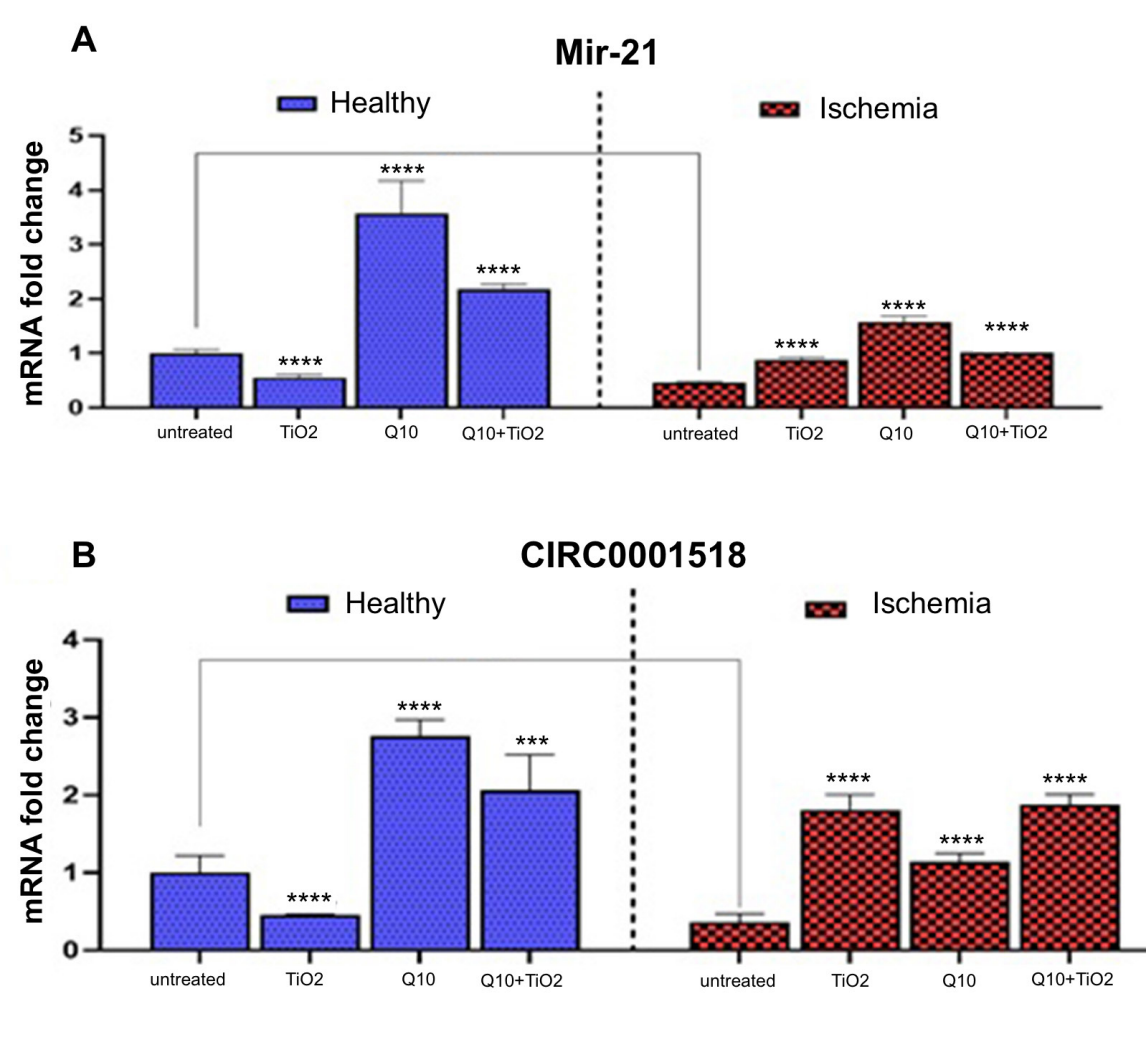
Figure 4. miR-21 (A) and circ0001518 (B) expressions in testicle cells of healthy and I/R rats that received CoQ10+TiO2-NPs. *** P<0.0001 and **** P<0.00001 vs. untreated rats.

Also, miR-21 and circ0001518 expressions upregulated in healthy rats that received CoQ10 and CoQ10 plus TiO2-NPs compared with healthy control rats (Figure-4). However, the administration of TiO2-NPs resulted in the downregulation of miR-21 and circ0001518 expressions. On the other hand, testicular I/R induction resulted in a nearly 2-fold reduction in miR-21 and circ0001518 expression levels (Figure-4).

Administration of TiO2-NPs, CoQ10, or their combination resulted in miR-21 and circ0001518 overexpression in testicular I/R rats compared to control testicular I/R rats (Figure-4).

As the showed in Table-2, the Bax expression had a significant negative correlation with the Bcl-2 (r=-0.804), miR-21 (r=-0.898), and circ0001518 (r=-0.712) expressions. In other words, with the upregulation of the Bax gene, we observed downregulations of the other genes. However, the Bcl-2 gene showed a significant positive correlation with miR-21 (r=0.703) and circ0001518 (r=0.772). There was a significant positive correlation between miR-21 expression and circ0001518 (r=0.594).

## Discussion

**Table:** Table-2. Correlational Analysis of Studied Genes

**Genes**	***BAX***	***BCL2***	***BAX-BCL2***	***miR21***	***Circ0001518***
***BAX***	1	-0.80418	0.93673	-0.89847	-0.71263
***BCL2***	-0.80418	1	-0.96137	0.70309	0.77274
***BAXBCL2***	0.93673	-0.96137	1	-0.83017	-0.78513
***miR21***	-0.89847	0.70309	-0.83017	1	0.59468
***CIRC0001518***	-0.71263	0.77274	-0.78513	0.59468	1

The present study evaluated the effects of CoQ10 and TiO2-NPs on spermatogenesis following testicular torsion/detorsion. In addition, the rate of apoptosis was assessed using flow cytometry and measuring genes expressions. Testicular I/R damage leads to the death of sperm cells, mostly due to a lack of oxygen supply for metabolic activity, depletion of cellular energy, and accumulation of toxic metabolites. In the reperfusion phase, increased production of ROS and reactive nitrogen species severely worsen ischemic damage in testicular tissue [20]. Accordingly, despite early diagnosis and clinical management, fertility disorders are the most important consequence of this type of testicular injury [21]. Some studies have shown severe degradation of sperm parameters following testicular I/R damage [21]. In the present study, testicular torsion/detorsion resulted in severe oligoasthenospermia.

We showed that CoQ10 administration improved sperm parameters in testicular I/R and healthy rats. CoQ10 has been shown to increase mitochondrial energy production by increasing ubiquinol (reduction form) and ubiquinone (oxide form) in seminal fluid and improving sperm parameters [10][22]. It can also effectively prevent DNA damage by reducing the level of ROS, which plays a crucial role in DNA damage [23]. Improvement of sperm parameters as a result of CoQ10 administration has been reported in other studies, which is similar to the findings of the present study [23]. In recent years, the green synthesis of NPs by plants has attracted much attention and can be considered an alternative to chemical methods [24]. Plants have antioxidant activity due to their secondary metabolites, such as phenol and flavonoids, which can prevent cell oxidative damage [25]. Therefore, plants have the potential to biodegrade ions and produce NPs that have antioxidant properties [26]. The biosynthesis of NPs through plants is a proper biocompatible method that is under consideration by researchers [27]. Numerous studies have been performed on the antioxidant properties of NPs and their ability to reduce apoptosis. In this regard, Taghizadeh et al. evaluated the antioxidant effects of selenium NPs on rat testicular tissue [28]. According to their results, selenium NPs have an antioxidant effect, increase superoxide dismutase, glutathione peroxidase, and testosterone levels, and reduce malondialdehyde [28].

In the present study, treatment with

TiO2-NPs in testicular I/R rats improved cell damage. Since the cells of the normal group were not under stress and had normal energy levels, it was likely that TiO2-NPs treatment caused stress and led to a severe decrease in internal energy and an increased rate of cell death. However, in the testicular I/R rats, the severe ischemic process caused a sharp drop in energy levels, and treatment with TiO2-NPs led to a slight decrease in internal energy levels; therefore, NPs in this group had antioxidant properties that led to reduced cell damage and death.

Oxidative stress induces apoptosis and reduces sperm quality, leading to the serious consequence of infertility in men [29]. ROS initiates a chain reaction by activating caspases that eventually lead to apoptosis [30]. The process of apoptosis removes unwanted or unnecessary cells in living organisms and interferes with many immune system mechanisms or diseases [30].

We observed that concomitant treatment with TiO2-NPs and CoQ10 following testicular I/R upregulated Bcl-2 gene expression, and as a result, it reduces apoptosis. Several models have been proposed for the ability of Bcl-2 to inhibit caspase cascade activ ation [31]. Bcl-2 prevents mitochondrial dysfunction, such as loss of potential membrane and membrane permeability transfer, and prevents the release of apoptogenic factors, such as cytochrome C and AIF via blocking membrane permeability. Bcl-2, on the other hand, interacts with the CED-4-LIKE protein and blocks caspases [32]. Also, Bcl-2 inhibits the independent release of cytochrome C [33] and the transfer of the BAX protein from the cytosol to the mitochondria [34]. The anti-apoptotic effects of CoQ10 and the decrease in the Bax/Bcl-2 expression ratio have been shown in other studies [35] and were attributed to the antioxidant properties of this compound and the increase in antioxidant enzymes [36]. In the present study, co-administration of CoQ10 and TiO2-NPs decreased the Bax/Bcl-2 expression ratio and apoptosis. The effects of NPs on seminal fluid and sperm quality appear to be concentration-dependent; therefore, to reduce the possible damage of NPs to the reprodu ctive system, the use of antioxidants such as CoQ10 is recommended.

The miR-21 is proposed to be an anti-apoptotic element that appears to inhibit caspase activity and regulate apoptosis by regulating the gene expressions of TNF-α, PTEN, RAS, and PI3K. Therefore, this element has been referred to as an oncogene in various cancers [37]. In the present study, testicular I/R significantly reduced miR-21 levels in rat testes cells. Administration of TiO2-NPs in normal rats significantly reduced the expression of miR-21 due to increases in oxidative stress and apoptosis. In the I/R group, it increased miR-21 expression. Administration of CoQ10 significantly upregulated miR-21 expression. CoQ10 reduced apoptosis by decreasing the Bax/Bcl-2 expression ratio and increasing miR-21 expression.

The results of studies show that there are 245 unique circRNAs in the male reproductive system [16][38]. KEGG studies indicate that these circRNAs originate from genes involved in stem cell differentiation, reproduction, and sex determination [16]. Therefore, circRNAs can be considered biomarkers for the evaluation of reproductive diseases [16].

Recent research has shown that circRNAs act as potential molecular markers for the diagnosis and treatment of diseases and plays important roles in the onset and progression of human diseases [39][40][41][42]. Some circRNAs appear to be involved in the regulation of apoptosis in cells. Endogenous circUBAP2 and hsa_circ_0001892 both compete for miR-143 and inhibit apoptosis in myeloma and osteosarcoma cells [39][43]. The anti-apoptotic effect of circRNA0001518 was confirmed in the current study because of the increase in apoptosis in testicular I/R cells and in the groups that received TiO2-NPs. In addition, following the administration of CoQ10 in both control and testicle I/R groups, we encountered an overexpression of circRNA0001518, which reaffirmed the anti-apoptotic effects of this circRNA. Our results indicate the beneficial effects of CoQ10 and TiO2-NPs in compensating for testicular I/R damage.

## Conclusion

It seems that CoQ10 along with

TiO2-NPs could reduce I/R testicular damage, and this was attributed to reductions in apoptosis, a decreased Bax/Bcl-2 expression ratio, and overexpression of miR-21 and circRNA0001518.

## Conflict of Interest

In this study, there is no financial support and/or conflict of interest.

## References

[R1] Sha J, Zhou Z, Li J, Yin L, Yang H, Hu G, et al (2002). Spermatogenesis study group . Identification of testis
development and spermatogenesis-related genes in human and mouse testes
using cDNA arrays. Mol Hum Reprod.

[R2] Omotehara T, Wu X, Kuramasu M, Itoh M (2020). Connection between seminiferous tubules and epididymal duct is
originally induced before sex differentiation in a sex-independent manner. Dev Dyn.

[R3] Darsini N, Hamidah B, Suyono SS, Ashari FY, Aswin RH, Yudiwati R (2019). Human Sperm Motility, Viability, and Morphology Decreased after
Cryopreservation. Folia Medica Indonesiana.

[R4] Paul C, Teng S, Saunders PT (2009). A single, mild, transient scrotal heat stress causes hypoxia and
oxidative stress in mouse testes, which induces germ cell death. Biol Reprod.

[R5] Farias JG, Puebla M, Acevedo A, Tapia PJ, Gutierrez E, Zepeda A, et al (2010). Oxidative stress in rat testis and epididymis under intermittent
hypobaric hypoxia: protective role of ascorbate supplementation. J Androl.

[R6] Aitken RJ (2017). Reactive oxygen species as mediators of sperm capacitation and
pathological damage. Mol Reprod Dev.

[R7] Arena S, Iacona R, Antonuccio P, Russo T, Salvo V, Gitto E, et al (2017). Medical perspective in testicular ischemia-reperfusion injury. Exp Ther Med.

[R8] Tuglu D, Yuvanc E, Yilmaz E, Gencay IY, Atasoy P, Kisa U, et al (2015). The antioxidant effect of dexmedetomidine on testicular
ischemia-reperfusion injury. Acta Cir Bras.

[R9] Al Saadi, Assaf Y, Farwati M, Turkmani K, Al-Mouakeh A, Shebli B, et al (2021). Coenzyme Q10 for heart failure. Cochrane Database Syst Rev.

[R10] Crane FL (2007). Discovery of ubiquinone (coenzyme Q) and an overview of
function. Mitochondrion.

[R11] Prakash S, Sunitha J, Hans M (2010). Role of coenzyme Q(10) as an antioxidant and bioenergizer in
periodontal diseases. Indian J Pharmacol.

[R12] Hussain M, Ceccarelli R, Marchisio D, Fino D, Russo N, Geobaldo F (2010). Synthesis, characterization, and photocatalytic application of
novel TiO2 nanoparticles. CEJ.

[R13] Hirst SM, Karakoti A, Singh S, Self W, Tyler R, Seal S, et al (2013). Bio-distribution and in vivo antioxidant effects of cerium oxide
nanoparticles in mice. Environ Toxicol.

[R14] Eriksson P, Tal AA, Skallberg A, Brommesson C, Hu Z, Boyd RD, et al (2018). Cerium oxide nanoparticles with antioxidant capabilities and
gadolinium integration for MRI contrast enhancement. Sci Rep.

[R15] Abdelazim AM, Saadeldin IM, Swelum AA, Afifi MM, Alkaladi A (2018). Oxidative Stress in the Muscles of the Fish Nile Tilapia Caused
by Zinc Oxide Nanoparticles and Its Modulation by Vitamins C and E. Oxid Med Cell Longev.

[R16] Liu KS, Pan F, Mao XD, Liu C, Chen YJ (2019). Biological functions of circular RNAs and their roles in
occurrence of reproduction and gynecological diseases. Am J Transl Res.

[R17] Elshaari F, Elfageih R, Sheriff D (2012). Testicular Torsion-Detorsion- Histological and Biochemical
Changes in Rat Testis. J Cytol Histol.

[R18] Entezari M, Sharifi ZN, Movassaghi S, Atabi F, Jamali Z, Salimi A (2019). Neuroprotective Effects of Cyperus rotundus Rhizome Extract on
Ischemic Brain Injury: Expression profile of Bax, Bcl2, Bad and Bclxl Genes. Drug & Advanced Sciences Journal.

[R19] Xu X, Kriegel AJ, Jiao X, Liu H, Bai X, Olson J, et al (2014). miR-21 in ischemia/reperfusion injury: a double-edged sword. Physiol Genomics.

[R20] Wilhelm Filho, Torres MA, Bordin AL, Crezcynski-Pasa TB, Boveris A (2004). Spermatic cord torsion, reactive oxygen and nitrogen species and
ischemia–reperfusion injury. Mol Aspects Med.

[R21] Dokmeci D (2006). Testicular torsion, oxidative stress and the role of antioxidant
therapy. Folia Med (Plovdiv).

[R22] Mancini A, Balercia G (2011). Coenzyme Q(10) in male infertility: physiopathology and therapy. Biofactors.

[R23] Lafuente R, González-Comadrán M, Solà I, López G, Brassesco M, Carreras R, et al (2013). Coenzyme Q10 and male infertility: a meta-analysis. J Assist Reprod Genet.

[R24] Iravani S (2011). Green synthesis of metal nanoparticles using plants. Green Chem.

[R25] Pourmorad F, Hosseinimehr S, Shahabimajd N (2006). Antioxidant activity, phenol and flavonoid contents of some
selected Iranian medicinal plants. AJB.

[R26] Santhoshkumar T, Rahuman AA, Jayaseelan C, Rajakumar G, Marimuthu S, Kirthi AV, et al (2014). Green synthesis of titanium dioxide nanoparticles using Psidium
guajava extract and its antibacterial and antioxidant properties. Asian Pac J Trop Med.

[R27] Dousti B, Nabipour F, Hajiamraei A (2019). Green Synthesis of Silver Nanoparticle Using Aqueous Extract of
Fumaria Parviflora and Study of its Antibacterial and Antioxidant Properties. RJMS.

[R28] Taghizadeh L, Eidi A, Mortazavi P, Rohani AH (2017). Effect of selenium on testicular damage induced by varicocele in
adult male Wistar rats. J Trace Elem Med Biol.

[R29] Sedha S, Kumar S, Shukla S (2015). Role of Oxidative Stress in Male Reproductive Dysfunctions with
Reference to Phthalate Compounds. Urol J.

[R30] Agarwal A, Sharma RK, Nallella KP, Thomas AJ, Alvarez JG, Sikka SC (2006). Reactive oxygen species as an independent marker of male factor
infertility. Fertil Steril.

[R31] Cheng EH, Kirsch DG, Clem RJ, Ravi R, Kastan MB, Bedi A, et al (1997). Conversion of Bcl-2 to a Bax-like death effector by caspases. Science.

[R32] Kowaltowski AJ, Vercesi AE, Fiskum G (2000). Bcl-2 prevents mitochondrial permeability transition and
cytochrome c release via maintenance of reduced pyridine nucleotides. Cell Death Differ.

[R33] Yang J, Liu X, Bhalla K, Kim CN, Ibrado AM, Cai J, et al (1997). Prevention of apoptosis by Bcl-2: release of cytochrome c from
mitochondria blocked. Science.

[R34] Murphy KM, Ranganathan V, Farnsworth ML, Kavallaris M, Lock RB (2000). Bcl-2 inhibits Bax translocation from cytosol to mitochondria
during drug-induced apoptosis of human tumor cells. Cell Death Differ.

[R35] Shahrooz R, Moloody-Tappe M, Razi M, Zarei L, Mohammadi V (2020). Investigation of the Effect of Coenzyme Q10 Supplementation on
Apoptosis Gene Expression and Oxidative Stress after Busulfan Injection in
Rats. Yafte.

[R36] El-Khadragy M, Al-Megrin WA, AlSadhan NA, Metwally DM, El-Hennamy RE, Salem FEH, et al (2020). Impact of Coenzyme Q10 Administration on Lead Acetate-Induced
Testicular Damage in Rats. Oxid Med Cell Longev.

[R37] Wang Y, Zhou S, Fan K, Jiang C (2019). MicroRNA-21 and its impact on signaling pathways in cervical
cancer. Oncol Lett.

[R38] Zare Z, Eimani H, Mohammadi M, Mofid M, Dashtnavard H (2010). The effect of orally administered L-carnitine on testis tissue,
sperm parameters and daily sperm production in adult mice. Yakhteh.

[R39] Zhong Y, Du Y, Yang X, Mo Y, Fan C, Xiong F, et al (2018). Circular RNAs function as ceRNAs to regulate and control human
cancer progression. Mol Cancer.

[R40] Abi A, Farahani N, Molavi G, Gheibi Hayat (2020). Circular RNAs: epigenetic regulators in cancerous and
noncancerous skin diseases. Cancer Gene Ther.

[R41] Ikebuaso AD, Yama OE, Duru FI, Oyebadejo SA (2012). Experimental Testicular Torsion in a Rat Model: Effects of
Treatment with Pausinystalia macroceras on Testis Functions. J Reprod Infertil.

[R42] Zang J, Lu D, Xu A (2020). The interaction of circRNAs and RNA binding proteins: An
important part of circRNA maintenance and function. J Neurosci Res.

[R43] Wang H, Zhou W, Zhang J, Li H (2019). Role of JNK and ERK1/2 MAPK signaling pathway in testicular
injury of rats induced by di-N-butyl-phthalate (DBP). Biol Res.

